# Validation of β-lactam minimum inhibitory concentration predictions for pneumococcal isolates with newly encountered penicillin binding protein (PBP) sequences

**DOI:** 10.1186/s12864-017-4017-7

**Published:** 2017-08-15

**Authors:** Yuan Li, Benjamin J. Metcalf, Sopio Chochua, Zhongya Li, Robert E. Gertz, Hollis Walker, Paulina A. Hawkins, Theresa Tran, Lesley McGee, Bernard W. Beall, on behalf of the Active Bacterial Core surveillance team

**Affiliations:** Respiratory Diseases Branch, Division of Bacterial Diseases, National Center for Immunization and Respiratory Diseases, Centers for Disease Control and Prevention, U.S. Department of Health and Human Services, Atlanta, GA 30329 USA

**Keywords:** *Streptococcus Pneumoniae*, β-lactam antibiotics, Minimum inhibitory concentration (MIC), Penicillin binding protein typing (PBP typing), Predictive modeling

## Abstract

**Background:**

Genomic sequence-based deduction of antibiotic minimum inhibitory concentration (MIC) has great potential to enhance the speed and sensitivity of antimicrobial susceptibility testing. We previously developed a penicillin-binding protein (PBP) typing system and two methods (Random Forest (RF) and Mode MIC (MM)) that accurately predicted β-lactam MICs for pneumococcal isolates carrying a characterized PBP sequence type (phenotypic β-lactam MICs known for at least one isolate of this PBP type). This study evaluates the prediction performance for previously uncharacterized (new) PBP types and the probability of encountering new PBP types, both of which impact the overall prediction accuracy.

**Results:**

The MM and RF methods were used to predict MICs of 4309 previously reported pneumococcal isolates in 2 datasets and the results were compared to the known broth microdilution MICs to 6 β-lactams. Based on a method that specifically evaluated predictions for new PBP types, the RF results were more accurate than MM results for new PBP types and showed percent essential agreement (MICs agree within ±1 dilution) >97%, percent category agreement (interpretive results agree) >93%, major discrepancy (sensitive isolate predicted as resistant) rate < 1.2%, and very major discrepancy (resistant isolate predicted as sensitive) rate < 1.4% for all 6 β-lactams. The identification of new PBP types over time was well approximated by a diminishingly increasing curve (Pearson’s *r* = 0.99) and minimally impacted overall MIC prediction performance.

**Conclusions:**

MIC prediction using the RF method could be an accurate alternative of phenotypic susceptibility testing even in the presence of previously uncharacterized PBP types.

**Electronic supplementary material:**

The online version of this article (doi:10.1186/s12864-017-4017-7) contains supplementary material, which is available to authorized users.

## Background

Sequence-based prediction of antimicrobial minimum inhibitory concentration (MIC) carries great potential to significantly improve clinical and public health microbiology [1, 2]. In clinical isolates of the pneumococcus, an important human pathogen, the primary determinants of β-lactam resistance are alterations in the transpeptidase domains (TPDs) of 3 critical penicillin-binding proteins (PBPs): PBP1a, PBP2b, and PBP2x [3–7]. We previously have developed a “PBP typing” system based on sequence signatures in the TPD of the 3 PBPs to track and predict β-lactam minimum inhibitory concentration (MIC) in pneumococci [8–10]. Two prediction models (MM and RF) were developed by using phenotypic MIC data as the response and the TPD amino acid sequences as the predictors. The trained models accurately deduced β-lactam MICs of a test isolate if it carried a PBP type that was included in the training dataset (characterized PBP types) [8–10]. For a new PBP type that was not included (uncharacterized PBP types), the predicted MICs were generally less accurate, yet the performance has not been fully evaluated [9].

All three *pbp* loci used in the PBP typing scheme have been shown to be hot spots of horizontal gene transfer in different pneumococcal lineages [11, 12]. Soon after the large-scale consumption of β-lactam antibiotics began, inter-species recombination between pneumococci and other more frequently colonizing *Streptococcus mitis* group members resulted in the transfer of many resistance-conferring *pbp* alleles into the pneumococcal population. [13–15]. Following subsequent mutation and intra-species recombination, a large number of *pbp* alleles associated with a wide range of β-lactam MICs were generated. Due to the high plasticity of pneumococcal genomes, it is unlikely that all possible PBP types will be documented. Thus, the overall accuracy of the sequence-based MIC prediction algorithms depends on both the probability of encountering new PBP types in a given population and the prediction performance for these new PBP types.

In this study we assessed the β-lactam MIC prediction models specifically for new PBP types using a novel method. The prediction models were further validated in an additional dataset from the same population. We also developed and validated a simple model to predict the growth rate of PBP types as more isolates are sequenced from the population. The results would address whether the predicted MICs can be an accurate alternative to phenotypic MICs even in the presence of previously uncharacterized PBP types.

## Methods

### Isolates and characterization

Isolates from two previously published datasets were used in this study. Dataset1 consisted of 2528 invasive pneumococcal isolates selected from the Active Bacterial Core surveillance (ABCs) over the years 1998–2015 (Additional file 1: Table S1) [9]. ABCs is an active, population-based and laboratory-based surveillance system that is part of the Centers for Disease Control and Prevention’s (CDC) Emerging Infections Program (See ABCs surveillance reports for population sizes, IPD incidence, antimicrobial susceptibility data and other information at http://www.cdc.gov/abcs/reports-findings/surv-reports.html). Dataset2 consisted of another 1781 invasive pneumococcal isolates selected from the ABCs in surveillance year 2015 (Additional file 2: Table S2) [10]. MICs for 6 β-lactam antibiotics, including penicillin (PEN), amoxicillin (AMO), meropenem (MER), cefotaxime (TAX), ceftriaxone (CFT) and cefuroxime (CFX), were determined with the broth microdilution method as previously described [16]. When MIC was analyzed as a numeric variable, an MIC of “= X” was treated as value X; an MIC of “<= X” was approximated as value X; and an MIC of “> X” was approximated as value 2X. A comparison of the Dataset1 and Dataset2 using t-SNE is shown in Additional file 3: Figure S1.

Genomic DNA samples from all isolates were sequenced as multiplexed libraries on the Illumina HiSeq or MiSeq platform to produce paired end reads [8, 10]. The Illumina short reads were analyzed by the CDC pneumococcal typing pipeline as described previously [8, 10] to extract the transpeptidase amino acid sequences of PBP1a, PBP2b, and PBP2x (Additional files 4 and 5: Tables S3 and S4). Each unique TPD amino acid sequence was assigned an identifier and the three-number combination from each isolate was assigned as the “PBP type”. All characterized PBP types and associated β-lactam MICs are publicly available at https://www.cdc.gov/streplab/mic-tables.html.

### β-lactam MIC prediction model

The previously described Mode MIC (MM) model and Random Forest (RF) model were evaluated in this study [9]. Briefly, based on the phenotypic MIC data and PBP type in a training dataset (Dataset1 was used as the training dataset for MIC predictions of Dataset2, see the “Leave-one-type-out cross validation” section below for the selection of training and testing dataset for Dataset1), the MM model assigned the highest MIC among the most frequently observed MIC(s) for a PBP type in the training dataset as the predicted MIC of a test isolate with the same PBP type. A new PBP type that was not seen in the training dataset was approximated by a training PBP type showing highest amino acid identity. In contrast, the RF model were trained by using the amino acid at each position of the 3 TPDs as predictors and the phenotypic MIC (log2 transformed) as a continuous response. The trained RF model then predicted the MIC of a test isolate based on its TPD amino acid sequence. For a given position in the TPDs, any amino acid not seen in the training dataset was approximated by training amino acid with the least BLOSUM62 [17] distance. The R package “randomForest” was used for RF model training and prediction. The number of trees was set as 5 times of the number of total training isolates following the recommendation of the software manual to ensure that every input row gets predicted several times. Other parameters were set as the default values, including number of variables randomly sampled as candidates at each split (mtry) = (number of predictors/3; 91 for Dataset1). RF model tuning on mtry was performed using the R package “caret”. Minimum variation of model performance around the chosen parameter values was observed (Additional file 6: Table S5). A 10-fold cross-validation of the RF model was also performed the R package “caret” on Dataset1 (Additional file 7: Table S6). The resulting R^2^ metrics showed a median of 0.97 (range, 0.94–0.98).

### Leave-one-type-out cross validation

Dataset1 was divided into training dataset and test dataset in the “leave-one-type-out” cross-validation in a special manner. For a given PBP type in Dataset1, all isolates of this PBP type were selected as the testing dataset while the remaining isolates were used as the training dataset. Thus, the testing dataset contains only a PBP type that was not included in the training dataset (new PBP type). The leave-one-type-out cross-validation specifically evaluated the prediction performance in a situation where a new PBP type was encountered. MICs in the test dataset were predicted using the MM and RF models parameterized by the training dataset. Partition of Dataset1 was repeated 307 times by using each PBP type in turn to select out the testing isolates. The predicted MICs were pooled together and compared against the phenotypic MICs. If an RF predicted MIC did not fall exactly on a two-fold dilution concentrations (e.g. 1, 0.5, 0.25……), the predicted MIC was log2 transformed and rounded to the nearest log2 transformed two-fold dilution concentration. A response randomization test was also performed on Dataset1. The PEN MIC labels were randomized over the input PBP sequences and the “leave-one-type-out” cross validation was performed based on the randomized Dataset1. After randomization, no correlation between the phenotypic log2 transformed MIC measurements and the predictions was observed (linear regression R^2^ = 0.032, Additional file 3: Figure S2).

### Evaluation of prediction performance

The predicted MICs were treated as results of a new method and the microdilution MICs results of a reference method. For each isolate, the predicted MIC was compared to the microdilution MIC for each antibiotic according to the FDA guidance for antimicrobial susceptibility test systems [18]. Briefly, the guidance’s definitions are:

- Essential Agreement (EA): Agreement within plus or minus one two-fold dilution of reference MIC;
- Category Agreement (CA): Agreement of interpretive results (Susceptible (S), Intermediate (I), or Resistant (R));
- major discrepancy (maj): The reference category result is S and the new method result is R; and very major discrepancy; and (vmj): The reference category result is R and the new method result is S.

MIC interpretive standards are shown in Table 1 and are consistent with CLSI stadards [19]. To demonstrate substantial equivalence between the reference and the new methods, criteria for acceptable performance include: 1) percent essential and category agreement >89.9%; 2) a maj rate of ≤3%; and 3) an upper 95% confidence limit for the true vmj rate of ≤7.5% and the lower 95% confidence limit for the true vmj ≤1.5%.

**Table 1:** Interpretive Standard for β-lactam antibiotics according to the CLSI form M100-S23

Antibiotics	Breakpoint MIC (μg/mL)		
	Susceptible (S)	Intermediate (I)	Resistant (R)
Penicillin (PEN)	≤0.06	0.12–1	≥2
Amoxicillin (AMO)	≤2	4	≥8
Meropenem (MER)	≤0.25	0.5	≥1
Cefotaxime (TAX)	≤0.5	1	≥2
Ceftriaxone (CFT)	≤1	2	≥4
Cefuroxime (CFX)	≤0.5	1	≥2

### Model of PBP type growth

To predict the number of new PBP types that will be found when sequencing additional isolates, we employed a simple power law-like model similar to the one for calculating bacterial pan-genome size [20–22]. We denoted *x* as the number of isolates sequenced and *y* as the number of PBP types found in these *x* isolates. For each one additional isolate sequenced, *x* increases by one and *y* either remains the same or increases by one depending on whether the additional isolate contains a new PBP type. The rate at which *y* increases as *x* increases was approximated by Eq. 1, where *a* and *b* are the two model parameters.

1 $$ \frac{dy}{dx}=a{x}^b $$

Given the initial condition (x = 1, y = 1), the solutions to Eq. 1 are

2 $$ y=\frac{a}{b+1}{x}^{b+1}+1-\frac{a}{b+1};b\ne -1 $$

3 $$ y= aln(x)+1;\kern0.5em b=-1 $$

With an infinite number of sequenced isolates, the number of PBP types will approach $\left(1-\frac{a}{b+1}\right)$ if *b* <  − 1; otherwise it will approach infinity. To estimate the model parameters, we generated 1000 permutations of the order of genome addition in Dataset1. In each permutation, the number of PBP types found on sequential addition of each new genome was fitted to Eqs. 2 and 3, respectively, using the Nonlinear Least Squares (nls) function implemented in the R software [23]. In a typical permutation, residual standard error of fitting Eq. 2 was about 20-fold smaller than that of fitting Eq. 3. We therefore selected Eq. 2 as the predictive model and obtained 1000 fitted *a* and *b* value combinations. To calculate the predicted value of *y* (*y*_*p*_) for a given *x* (*x*_*p*_), the *x*_*p*_ value was applied to Eq. 2 together with each of the fitted *a* and *b* combination to obtain 1000 *y* values, the median of which was used as *y*_*p*_. Prediction intervals (95% CI) for *y*_*p*_ were calculated based on the 2.5 and 97.5 percentile of these 1000 *y* values. Similarly, the point estimate and 95% CI for $\frac{dy}{dx}$ at a given *x* was calculated using the 1000 *a* and *b* values.

We also analyzed the number of PBP types using the maximum-likelihood estimator approach [24, 25] implemented in the “poweRlaw” package of the R package. The number of strains and the number of PBP types exhibited an approximately linear relationship on a log-log plot over more than two orders of magnitude in both the horizontal and vertical axes (Additional file 3: Figure S3). Parameter estimations from the “poweRlaw” package are shown in Additional file 3: Figure S4. A goodness-of-fit value of 0.13 and a bootstrapping hypothesis test *p*-value of 0.12 was observed.

### Statistics

Confidence interval for a proportion estimate was constructed using the exact binomial method (binom.test in R). All statistical analyses were performed in R version 3.2.2 [23]; graphics were also created in R 3.2.2.

## Results

### “Leave-one-type-out” evaluation of β-lactam MIC prediction for new PBP types

**Fig. 1:**
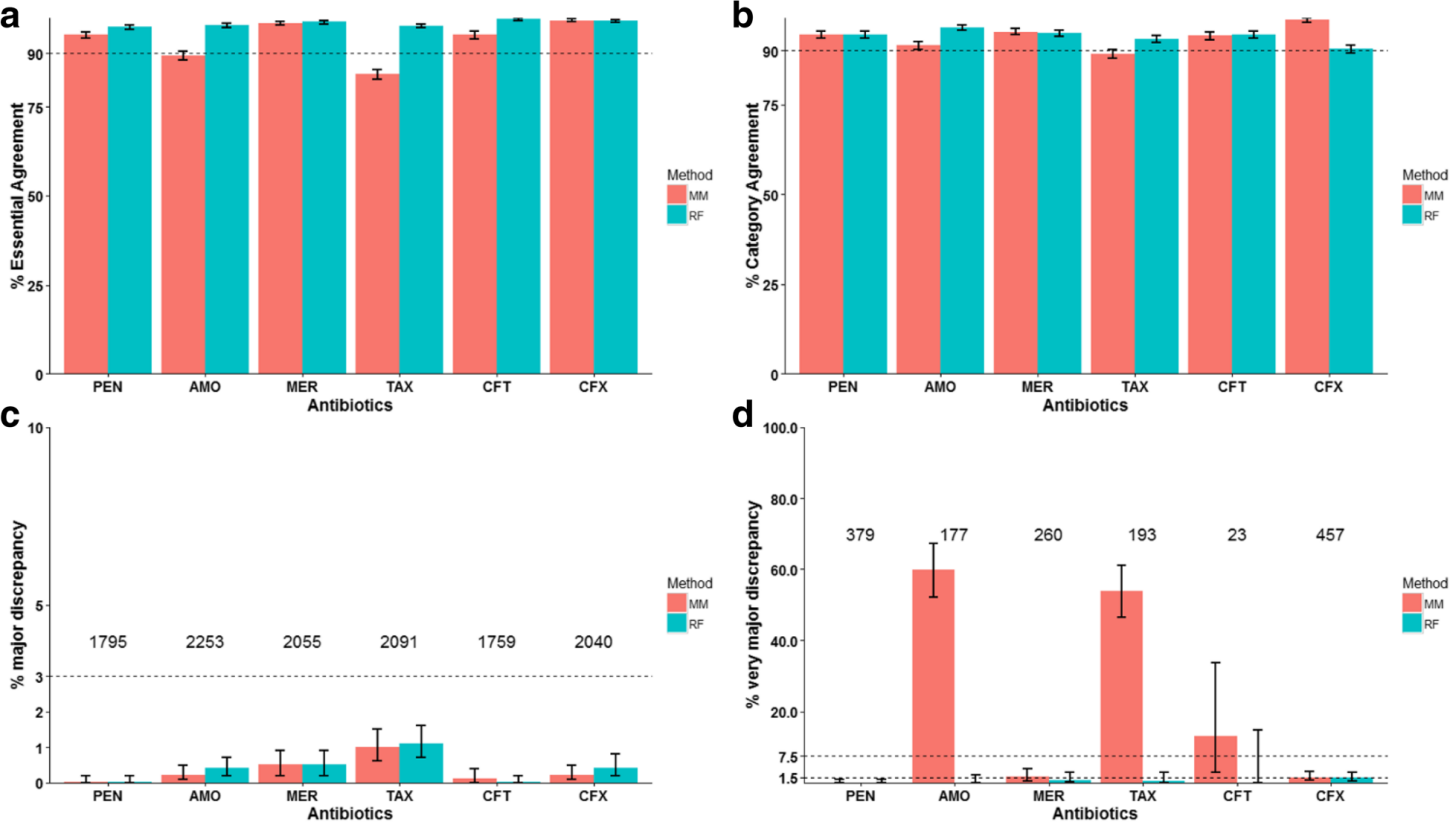
Comparison of the predicted β-Lactam MICs with phenotypic MICs specifically for “new” PBP types using the “leave-one-type-out” cross validation of Dataset1. All isolates of a PBP type were selected as the testing data and the remaining isolates were used as the training data. The MM (*red*) and RF (*green*) models were parametrized by the training data and then used to predict the MIC of the testing isolates, which represent a PBP type that was not included in the training data. The procedure was applied to each PBP type in turn, resulting in predicted MICs for each isolate in Dataset1, which were compared with the phenotypic MICs. **a** Percent Essential Agreement (MICs agree within ±1 2-fold dilution) between the predicted and phenotypic MICs. **b** Percent Category Agreement (interpretive results agree) between the predicted and phenotypic MICs. **c** Rate of major discrepancy (phenotypically sensitive isolate predicted as resistant). The number of phenotypically sensitive isolates that was used to calculate this rate is shown above the corresponding antibiotic. **d** Rate of very major discrepancy (phenotypically resistant isolate predicted as sensitive). The number of phenotypically resistant isolates that was used to calculate this rate is shown above the corresponding antibiotic. *Error bars* are 95% confidence intervals. PEN: penicillin; AMO: amoxicillin; MER: meropenem; TAX: cefotaxime; CFT: ceftriaxone; CFX: cefuroxime

We designed a “leave-one-type-out” cross validation following the principle of the standard “leave-one-out” cross validation to evaluate prediction performance specifically for new PBP types (see Methods for detail). Applying the “leave-one-type-out” approach to the 2528 isolates in Dataset1, which included 59 serotypes and 403 MLSTs, we calculated the EA, CA, maj and vmj for each of the 6 β-lactam antibiotics (Fig. 1). Results from the RF models showed higher than 97% EA for all 6 antibiotics, while results from the MM models showed EA lower than 90% for AMO and TAX (Fig. 1a). RF and MM models showed similar percent CAs and only the CA for TAX based on the MM model predictions was lower than 90% (Fig. 1b). Both RF and MM methods generated similar maj rates that were below 3% (Fig. 1c). The largest difference between the two models was the vmj rates (Fig. 1d). Results from the MM model showed unacceptably high vmj rates for 4 of the 6 antibiotics, with 3 of them higher than 50% (Fig. 1d). This was largely due to incorrect MM predictions for a considerable number of isolates with PBP type 13–11-16, which indicated a composite TPD amino acid sequence pattern of PBP1a-13, PBP2b-11, and PBP2x-16. The closely related PBP type 13–11-127, which was associated with substantially lower β-lactam MICs than 13–11-16, was used by the MM model for MICs of the PBP type 13–11-16 isolates. In contrast, results from the RF model showed acceptable vmj rates (lower 95% CI ≤1.5% and upper 95% CI ≤7.5%) for all 6 antibiotics. Given that vmj is a more serious discrepancy that should be minimized, the results indicated that β-lactam MIC predictions based on the RF model were more suitable for a new PBP type. In summary, the evaluation of prediction performance for new PBP types suggested that MM model-predicted MICs satisfied the three criteria of acceptable performance for PEN, MER, and CFX, while the RF model-predicted MICs satisfied the three criteria of acceptable performance for all six β-lactam antibiotics examined.

### PBP type growth approximated by a power law-like model

**Fig. 2:**
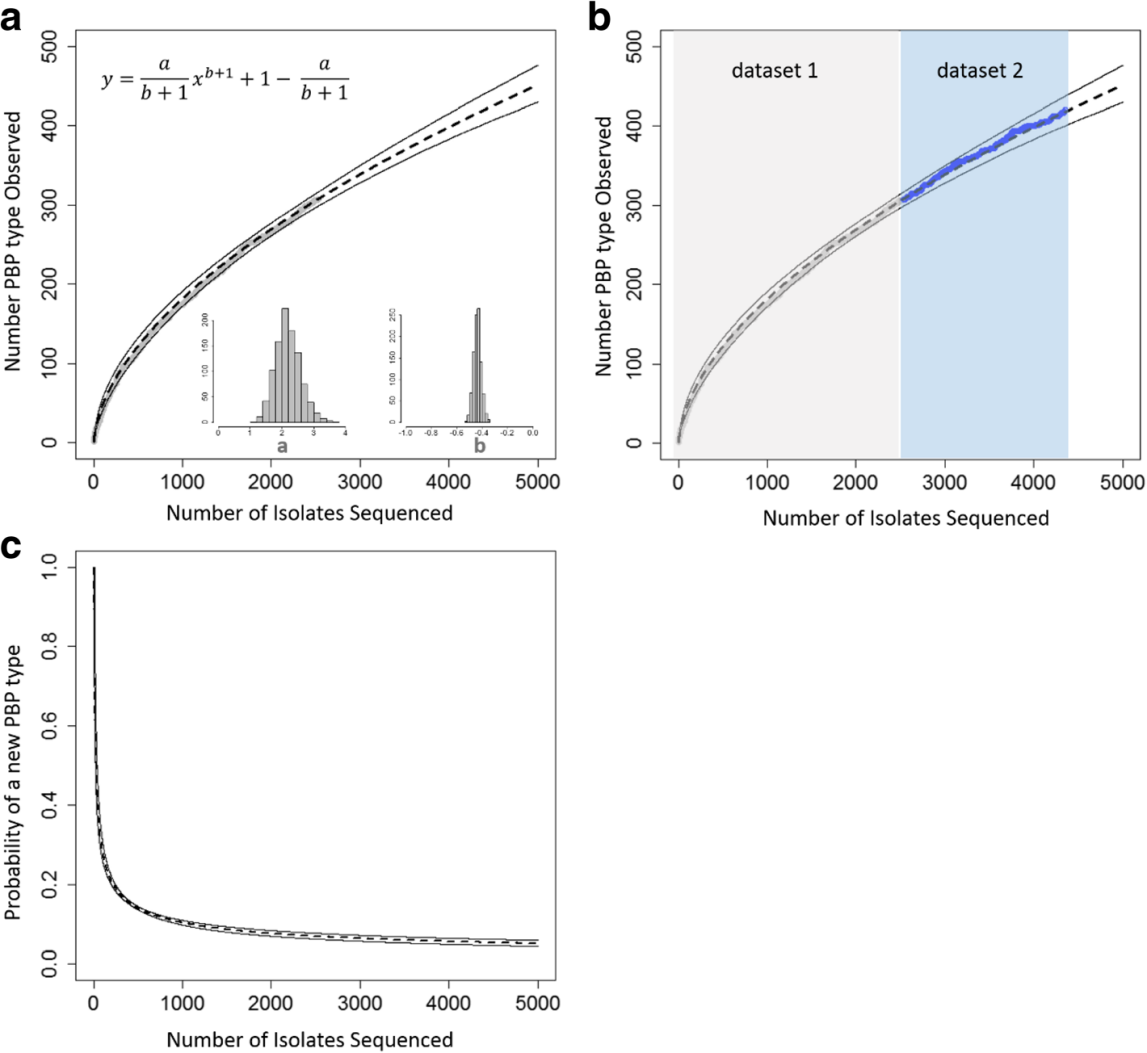
Model of the number of PBP types observed as a function of the number of isolates sequenced. **a** The *grey curve* indicates the observed number of PBP types on sequential addition of each new genome in Dataset1. The order of genome addition underwent 1000 permutations and data from each permutation were used to fit Eq. 2 (also shown in the figure) to estimate the model parameters “a” and “b” using nonlinear least squares regression. A histogram of the 1000 estimated “a” values and a histogram of the 1000 estimated “b” values are shown inside the figure. The *dashed curve* indicates the predicted number of PBP types at a given number of isolates sequenced. *Solid curves* indicate the prediction intervals. **b** Agreement between the predicted number of PBP types (*dashed curve*, same as in (**a**)) and the observed numbers (*blue curve*) on sequential addition of each isolate in Dataset2. **c** Diminishing probability of encountering a new PBP type as more isolates being sequenced. The *dashed curve* indicates the predicted probability of an additional isolate carrying a new PBP type at a given number of isolates sequenced (Eq. 1). *Solid curves* are the prediction intervals

The number of PBP types observed as a function of the number of isolates sequenced was approximated by a two-parameter power law-like model for the growth rate (Eq. 1 in Methods). For isolates in Dataset1, the order of addition was permutated and fitted to the Eq. 2 model using nonlinear least squares regression. The fitted values of Eq. 2 well approximated the observed increase of PBP types (Fig. 2a) in Dataset1 with a typical residual standard error of 2.41 on 2526 degrees of freedom. Based on 1000 permutations, the likely values of the two model parameters, *a* and *b*, were estimated (Fig. 2a). The estimated parameter *b* showed a mean of −0.44 (Fig. 2a), indicating a general trend of decreasing probability of encountering new PBP types. Caution should be taken in interpreting the parameter values as the maximum-likelihood estimator approach showed a low goodness-of-fit (Additional file 3: Figure S4).

Next, we validated the predicted growth of PBP types using Dataset2 (Fig. 2b). The observed number of PBP types on sequential addition of each isolate in Dataset2 (Fig. 2b, blue curve) were overlaid on the extrapolation of predictions based on Dataset1 (Fig. 2b, dashed curve) and a very good agreement was observed (Fig. 2b). For a typical permutation of Dataset2, the predicted and the observed number of PBP types showed a Pearson correlation coefficient of 0.99. Since both Dataset1 and Dataset2 represented invasive pneumococcal isolates from the ABCs surveillance areas, it appeared Eq. 1 would be good approximation for the growth rate of PBP types as we sequence more isolates from this population. Equation 1 gives the probability of encountering an isolate with a new PBP type given *x* isolates have been sequenced. Based on the estimated *a* and *b* values, the probability of encountering an additional isolate carrying a new PBP was approximately 0.072 (95% CI 0.062 to 0.076) after sequencing 2528 isolates (Dataset1) and 0.054 (95% CI 0.047 to 0.062) after sequencing 4309 isolates (Dataset1 and Dataset2).

### Minimal impact of new PBP types on the overall prediction accuracy

**Fig. 3:**
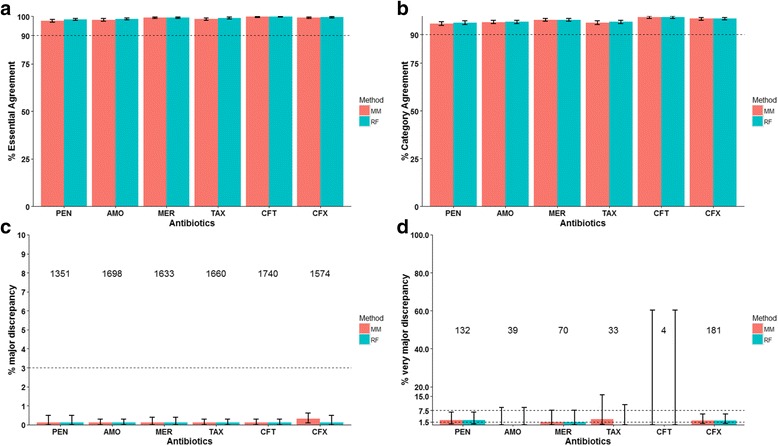
Comparison between the predicted β-Lactam MICs and phenotypic MICs for all isolates in Dataset2. PBP sequence and phenotypic MIC data of Dataset1 were used to train the MM (*red*) and RF (*green*) models. The trained models were used to predict MICs of all Dataset2 isolates. The resulting predicted MICs were compared with the phenotypic MICs of Dataset2 isolates. **a** Percent Essential Agreement (MICs agree within ±1 2-fold dilution) between the predicted and phenotypic MICs. **b** Percent Category Agreement (interpretive results agree) between the predicted and phenotypic MICs. **c** Rate of major discrepancy (phenotypically sensitive isolate predicted as resistant). The number of phenotypically sensitive isolates that was used to calculate this rate is shown above the corresponding antibiotic. **d** Rate of very major discrepancy (phenotypically resistant isolate predicted as sensitive). The number of phenotypically resistant isolates that was used to calculate this rate is shown above the corresponding antibiotic. *Error bars* are 95% confidence intervals. PEN: penicillin; AMO: amoxicillin; MER: meropenem; TAX: cefotaxime; CFT: ceftriaxone; CFX: cefuroxime

Modeling of PBP type growth suggested that the likelihood of encountering an additional isolate carrying a new PBP was small after we sequenced the 2528 isolates in Dataset1. In addition, β-lactam MIC prediction for new PBP types by RF model appeared to have good accuracy. We therefore hypothesized that the RF model trained by Dataset1 can accurately predict β-lactam MICs of Dataset2 isolates even in the presence of new PBP types. To test this hypothesis, PBP sequence and phenotypic MIC data of Dataset1 were used to train the RF model. For comparison purpose, we also trained the MM model using the same dataset. The trained models were used to predict MICs of Dataset2 isolates as the test dataset, which included 44 serotypes and 299 MLSTs. The resulting predicted MICs were compared with the phenotypic MICs (Fig. 3). The EA for 6 antibiotics ranged from 97.6% (PEN, MM method) to 99.8% (CFT, both methods) and no substantial difference between the two methods were observed (Fig. 3a). Similarly, the CA for 6 antibiotics ranged from 95.7% (PEN, MM method) to 99.1% (CFT, both methods). The maj rate calculation was based on the number of susceptible isolates for each antibiotic, which ranged from 1351 to 1740 (Fig. 3c). Neither RF nor MM method resulted in more than 4 maj events for any antibiotic (Fig. 3c). For PEN and CFX, both RF and MM predictions showed acceptable vmj rates (Fig. 3d, lower 95% CI ≤1.5% and upper 95% CI ≤7.5%). Estimation of vmj rates for AMO, and CFT was limited by small number (39 and 4, respectively; Fig. 3d) of resistant isolates, although no maj event was observed. In summary, the prediction performance of RF and MM methods were similar based on a representative testing dataset containing new PBP types. The predicted β-lactam MICs appeared to be essentially equivalent to the results of phenotypic testing. For the RF model, the positive predictive value (PPV) for β-Lactam resistance ranged from 0.80 to 0.96, and the negative predictive value (NPV) ranged from 0.98 to 1 (Additional file 8: Table S7).

**Fig. 4:**
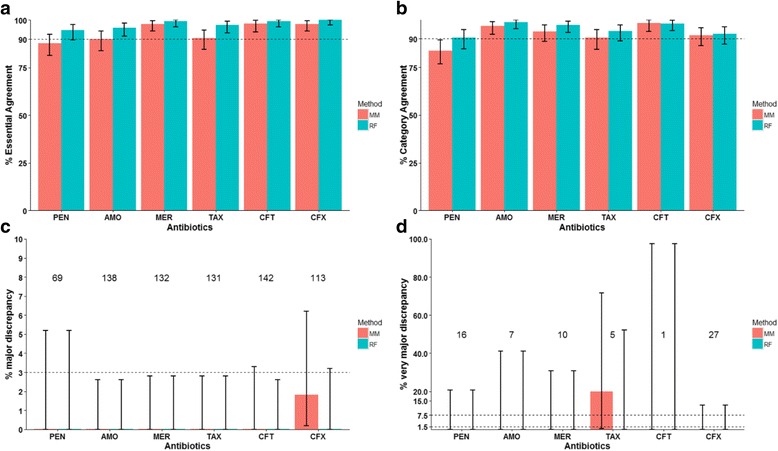
Comparison between the predicted β-Lactam MICs and phenotypic MICs for new PBP type in Dataset2. All isolates of Dataset1 were used to train the MM (*red*) and RF (*green*) models. The trained models were used to predict MICs of Dataset2 isolates that carried a PBP type not seen in Dataset1 (new PBP type). The resulting predicted MICs were compared with the phenotypic MICs of these Dataset2 isolates. **a** Percent Essential Agreement (MICs agree within ±1 2-fold dilution) between the predicted and phenotypic MICs. **b** Percent Category Agreement (interpretive results agree) between the predicted and phenotypic MICs. **c** Rate of major discrepancy (phenotypically sensitive isolate predicted as resistant). The number of phenotypically sensitive isolates that was used to calculate this rate is shown above the corresponding antibiotic. **d** Rate of very major discrepancy (phenotypically resistant isolate predicted as sensitive). The number of phenotypically resistant isolates that was used to calculate this rate is shown above the corresponding antibiotic. *Error bars* are 95% confidence intervals. PEN: penicillin; AMO: amoxicillin; MER: meropenem; TAX: cefotaxime; CFT: ceftriaxone; CFX: cefuroxime

Next, we focused on the 148 isolates in Dataset2 that carried 109 PBP types not present in Dataset1 (Fig. 4, Additional file 2: Table S2). For these new PBP types, the RF method produced noticeably higher EA and CA than the MM method (Fig. 4a, b), especially in the case of PEN, AMO, and TAX. In fact, percent EA and CA associated with the RF method was above 90% for all 6 antibiotics while this was not the case for the MM method. The RF model-predicted MICs showed no maj or vmj event for any of the 6 antibiotics (Fig. 4c, d). In contrast, the MM model-predicted MICs showed two maj events (both for CFX) and one vmj event for TAX (Fig. 4c, d). The results supported the conclusion of the previous “leave-one-type-out” cross validation that the RF model allows more accurate β-lactam MIC predictions than the MM model for a new PBP type.

## Discussions

Previous studies have demonstrated accurate sequence-based β-lactam MIC predictions for pneumococcal isolates carrying a characterized PBP type (phenotypic β-lactam MICs available for at least one isolate of this PBP type) [8, 9]. Here we systematically evaluated the prediction performance for uncharacterized PBP types (no phenotypic β-lactam MICs available) in a population. We compared two previously defined β-lactam MIC prediction models, MM and RF, and found the RF model delivered better prediction results for new PBP types than the MM model. A possible reason is that the MM model depended only on the most closely related PBP type that have been characterized to make predictions for a new PBP type, thus did not fully use the information in the training dataset. For example, a single amino acid change (PBP2x I371T) in PBP type 13–11-127 resulted in a commonly observed PBP type (13–11-16) that was associated with 4-fold higher PEN and AMO MICs compared to the original PBP type. The MM method simply used the MICs of the former as the MICs of the latter, resulting in high error rates. In contrast, the RF model accurately predicted the MICs for PBP type 13–11-16 as a new PBP type, because the same PBP2x I371T substitution occurred in other PBP types and the associated MIC changes have been used to train the RF model. The RF model could also be vulnerable to prediction errors if a new PBP type contained amino acid substitutions not occurring in any previously characterized PBP types, although such a new PBP type would become increasingly uncommon as we continue to expand the characterized PBP type database. Nonetheless, the empirical nature of the sequence-based MIC predictions stresses the importance of obtaining phenotypic MICs for new PBP types that contain unusual substitutions.

We also modeled the probability of encountering new PBP types in a given population because this factor will also impact the overall prediction accuracy. Using invasive pneumococcal isolates from the ABCs surveillance areas, we observed that the rate at which new PBP types was discovered was well approximated by a power law-like Eq. 2. Model fitting suggests that while the total number of PBP types is unlikely to plateau soon, as we sequence more isolates from ABCs surveillance areas, the growth rate is diminishing. This observation could reflect that the rate at which new PBP types emerge in this population is much slower than the rate at which new PBP types are detected because we have examined a large number of pneumococcal genomes from this population in a short period (approximately 4500 genomes in 2 years). Effectively the PBP types observed represented a subset of the finite, although large, number of PBP types in the current, largely static population structure. In the longer term, it is conceivable that genetic drift, selection, and migration would allow more PBP types to emerge, and the antibiotic selective pressure could lead to a more drastic change in the PBP that is not accounted for in the current model. Here we aimed to conduct an empirical model fitting to estimate the increase of PBP types in the near future where no dramatic change of antibiotic selection pressures was expected. More complicated models are possible although the simple approximation by Eq. 2 provided a good prediction for an independent test dataset (Fig. 2b). It should also be noted that the problem of potentially spurious power-law relationships could result in difficulties in statistical inference [24–26] and should be a priority of future research.

Although the MM method generated less accurate MIC predictions than the RF method for new PBP types, the overall prediction performance of the two methods for an independent Dataset2 was similar. This was because only a small proportion of the Dataset2 isolates carry a new PBP type (8.3%) and because MICs of the any PBP type were similar to those of a closely related PBP type in the training dataset (Dataset1). Combining Dataset1 and Dataset2 increased the number of characterized PBP types from 307 to 420 and will allow more accurate β-lactam MIC predictions for invasive pneumococcal isolates from the ABCs surveillance areas going forward. However, due to the high diversity of pneumococcal genomes worldwide, it is important to further evaluate the performance of PBP type-based MIC prediction in pneumococcal isolates from populations outside the U.S. and to document the PBP types causing β-lactam resistance in different countries. In addition, analysis of the constraints on evolutionary pathways leading to increased β-lactam MICs may provide insights into resistance mechanisms and help design better prediction models. The BLOSUM62 substitution was used as interpolate for unseen amino acids, which may not capture the biological effects of a substitution on β-lactam resistance. Incorporating the biochemical properties of amino acid, including charge, polarity, side chain length, and the R-group size, could be of value in improving prediction accuracy. Such limitation also highlights the importance of obtaining phenotypic MICs for newly found PBP types and to experimentally determine the specific contribution of an amino acid substitution to β-lactam resistance in future research.

## Conclusions

Predictions of pneumococcal β-lactam MICs using the RF method could be an accurate alternative of phenotypic susceptibility testing even in the presence of previously uncharacterized PBP types. The findings may lead to the development of sequencing-based extraction of antibiotic resistance information from clinical specimens without culturing, holding great promise to improve clinical and public health microbiology.

## Additional files

Additional file 1: Table S1. List of 2528 Dataset1 isolates. (CSV 254 kb)

Additional file 2: Table S2. List 1781 Dataset2 isolates. (CSV 260 kb)

Additional file 3: Figure S1. A comparison of the Dataset1 (DS1, red) and Dataset2 (DS2, green) samples using t-SNE. Individual amino acid positions that were used to define PBP type were used as the starting dimensions. **Figure S2.** A randomization test of the “leave-one-type out” cross-validation. (A) The MIC labels were randomized over the input PBP sequences before the “leave-one-type out” cross-validation using the RF method. MIC values were log2 transformed and rounded to the nearest integer. Correlation between the true penicillin MIC (Log2_PEN) and predicted MIC (Log2_PEN_RANDtest) is shown. A small amount of random variation to the location of each point was added to aid visualization. Adjusted R2 from a linear regression are shown on top. (B) Results from the “leave-one-type out” cross-validation in which the true MIC labels were used. **Figure S3.** Log-log plot of the number of strains (nStrain) and the number of PBP types (nPT) for the 4309 strains and 417 PBP types observed in Dataset1 and Dataset2. Redline indicates the fitted line of a linear regression. R2 estimation of the linear regression are shown on top. **Figure S4.** Results from the bootstrap procedure for the power law model. The dashed-lines give approximate 95% confidence intervals. (PDF 324 kb)

Additional file 4: Table S3. Transpeptidase domain (TPD) amino acid residuals of the 3 penicillin-binding proteins (PBPs) for Dataset1 isolates. (CSV 9068 kb)

Additional file 5: Table S4. Transpeptidase domain (TPD) amino acid residuals of the 3 penicillin-binding proteins (PBPs) for Dataset1 isolates. (CSV 6391 kb)

Additional file 6: Table S5. Results of tuning RF model parameter mtry, number of variables randomly sampled as candidates at each split. (CSV 430 bytes)

Additional file 7: Table S6. Results of a 10-fold cross-validation of the RF model. (CSV 1009 bytes)

Additional file 8: Table S7. The positive predictive value (PPV) and negative predictive value (NPV) for β-Lactam resistance. (CSV 569 bytes)

## Abbreviations

ABCs: Active Bacterial Core surveillance
AMO: Amoxicillin
BLOSUM: BLOcks SUbstitution Matrix
CA: Category Agreement
CDC: Centers for Disease Control and Prevention
CFT: Ceftriaxone
CFX: Cefuroxime
EA: Essential Agreement
maj: major discrepancy
MER: Meropenem
MIC: Minimum inhibitory concentration
MM: Mode MIC
PBP: Penicillin Binding Protein
PEN: Penicillin
RF: Random Forest
TAX: Cefotaxime
TPD: Transpeptidase domain
vmj: very major discrepancy
