# Supplementary material for: Validation of β-lactam minimum inhibitory concentration predictions for pneumococcal isolates with newly encountered penicillin binding protein (PBP) sequences
Source: BMC Genomics. 2017 Aug 15;18:621. doi: 10.1186/s12864-017-4017-7 (PMC5558719; doi:10.1186/s12864-017-4017-7)
Supplement: Supplementary file 3 — A comparison of the Dataset1 (DS1, red) and Dataset2 (DS2, green) samples using t-SNE. Individual amino acid positions that were used to define PBP type were used as the starting dimensions. Figure S2. A randomization test of the “leave-one-type out” cross-validation. (A) The MIC labels were randomized over the input PBP sequences before the “leave-one-type out” cross-validation using the RF method. MIC values were log2 transformed and rounded to the nearest integer. Correlation between the true penicillin MIC (Log2_PEN) and predicted MIC (Log2_PEN_RANDtest) is shown. A small amount of random variation to the location of each point was added to aid visualization. Adjusted R2 from a linear regression are shown on top. (B) Results from the “leave-one-type out” cross-validation in which the true MIC labels were used. Figure S3. Log-log plot of the number of strains (nStrain) and the number of PBP types (nPT) for the 4309 strains and 417 PBP types observed in Dataset1 and Dataset2. Redline indicates the fitted line of a linear regression. R2 estimation of the linear regression are shown on top. Figure S4. Results from the bootstrap procedure for the power law model. The dashed-lines give approximate 95% confidence intervals. (PDF 324 kb) [file 12864_2017_4017_MOESM3_ESM.pdf]

Figure S1

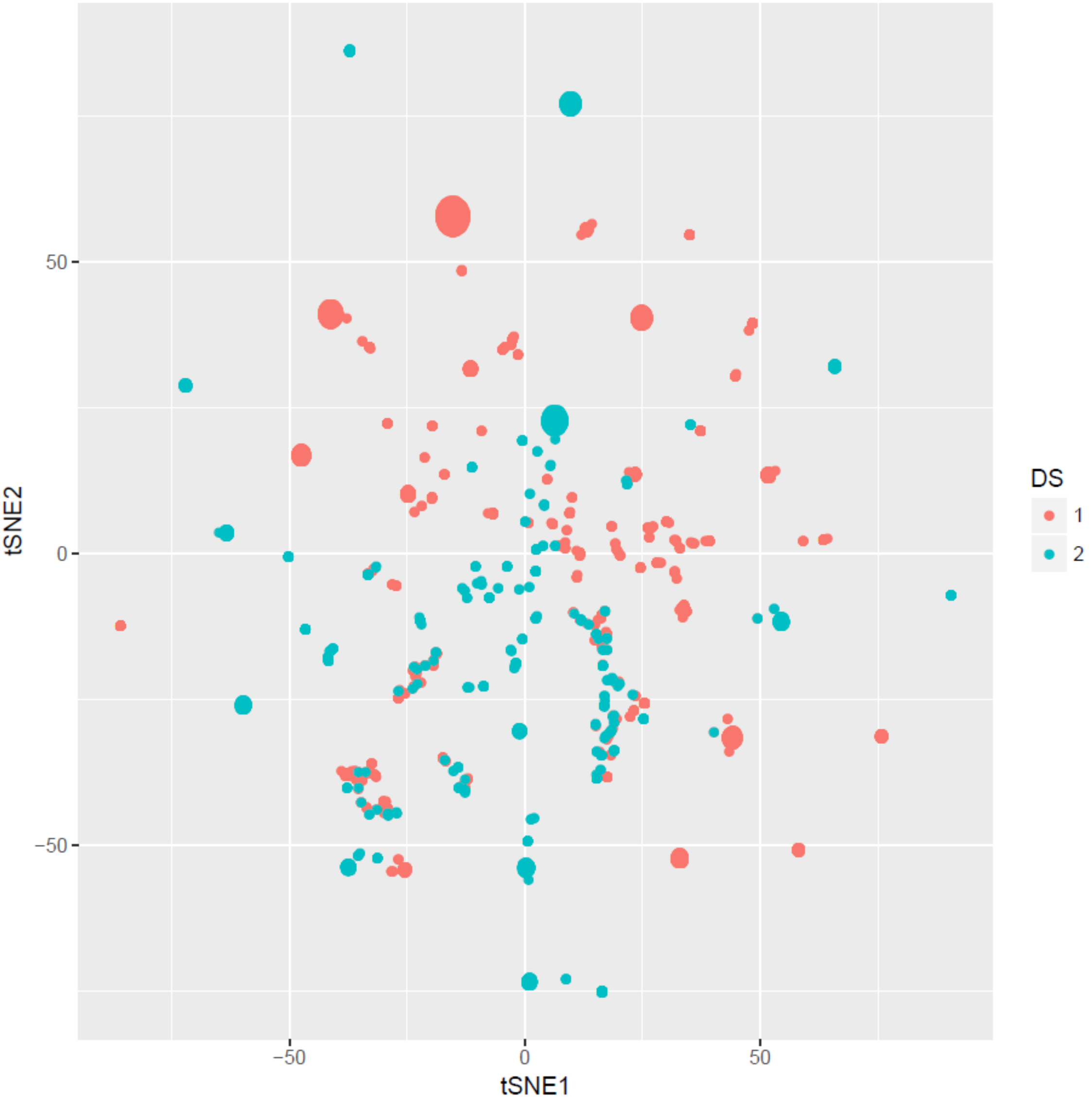

Figure S1. A comparison of the Dataset1 (DS1, red) and Dataset2 (DS2, green) samples using t-SNE. Individual amino acid positions that were used to define PBP type were used as the starting dimensions.

Figure S2

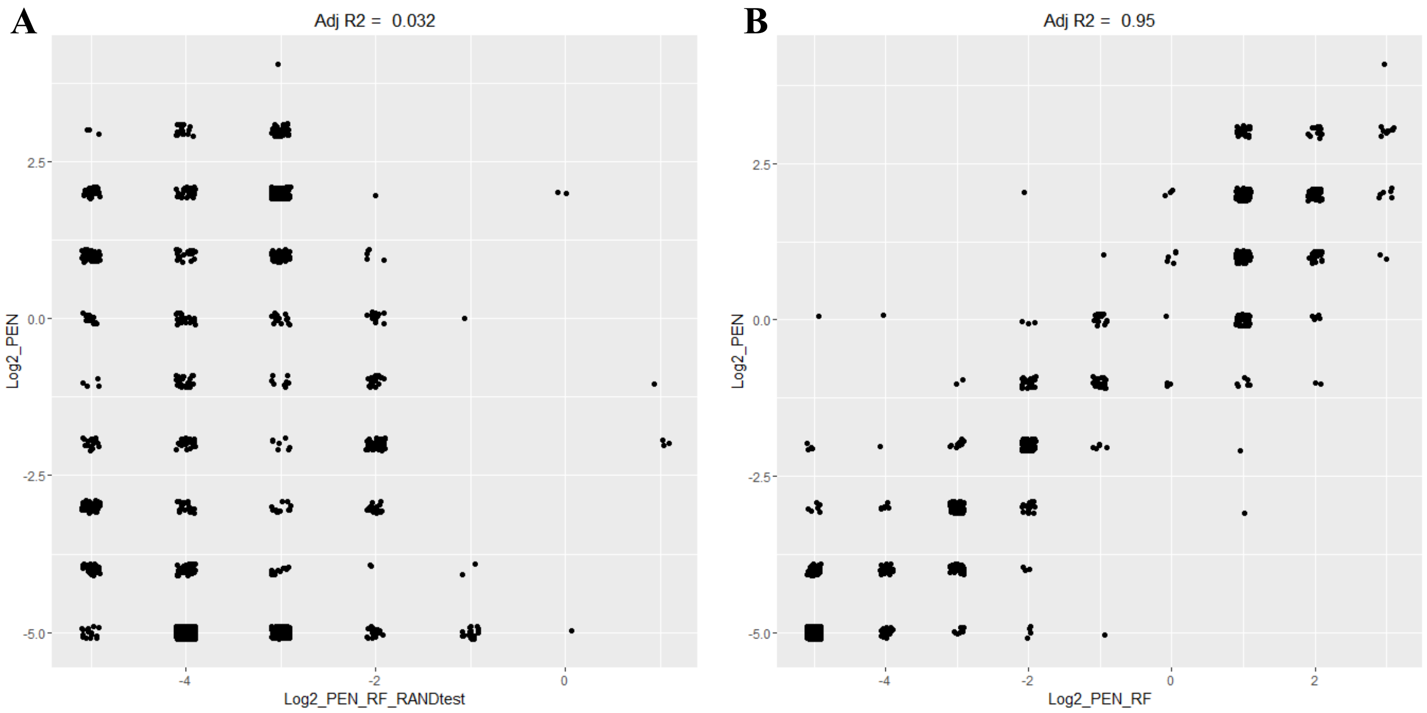

Figure S2. A randomization test of the "leave-one-type out" cross-validation. (A) The MIC labels were randomized over the input PBP sequences before the "leave-one-type out" cross-validation using the RF method. MIC values were log2 transformed and rounded to the nearest integer. Correlation between the true penicillin MIC (Log2_PEN) and predicted MIC (Log2_PEN_RANDtest) is shown. A small amount of random variation to the location of each point was added to aid visualization. Adjusted R^2 from a linear regression are shown on top. (B) Results from the "leave-one-type out" cross-validation in which the true MIC labels were used.

Figure S3

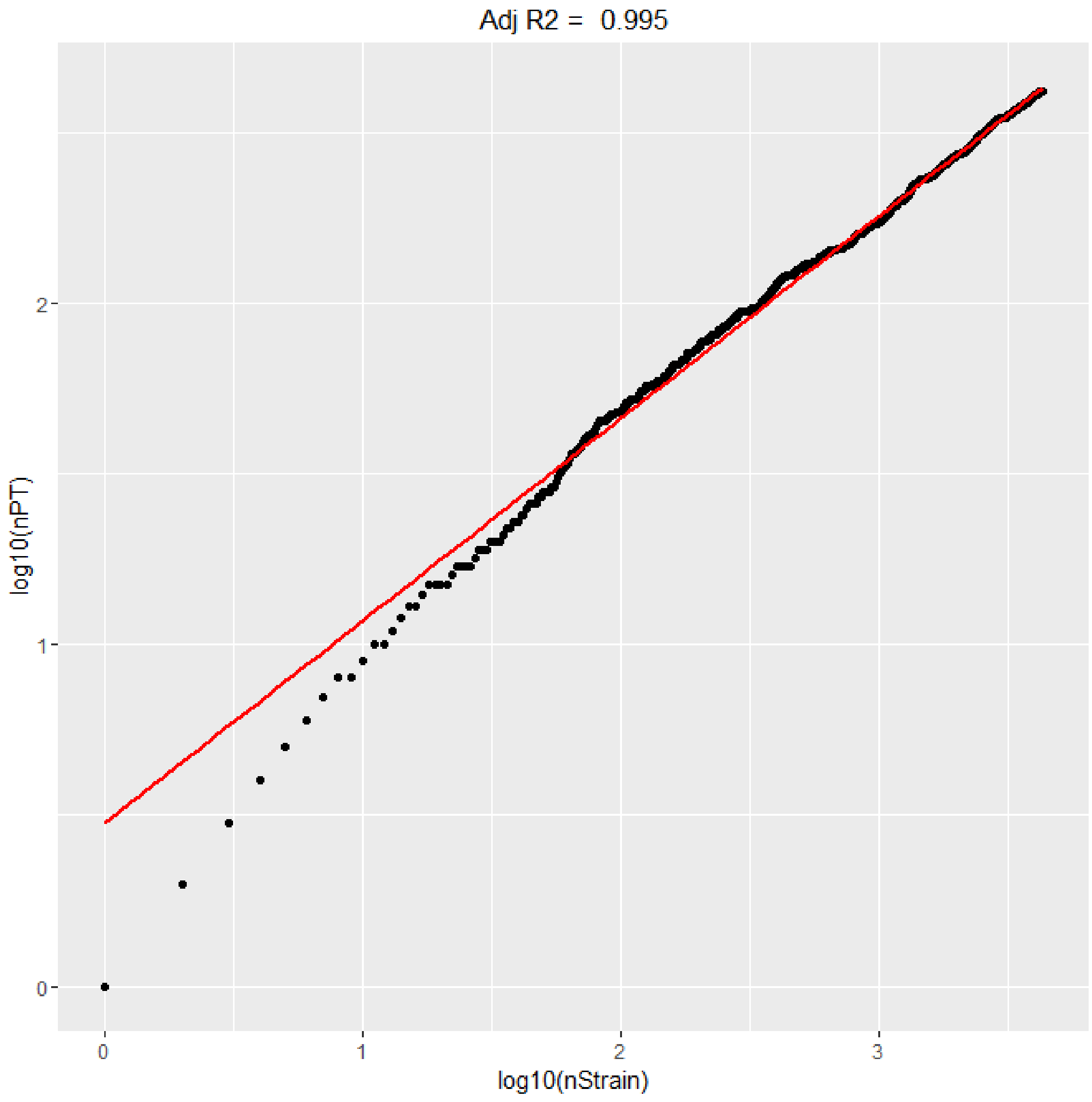

Figure S3. Log-log plot of the number of strains (nStrain) and the number of PBP types (nPT) for the 4309 strains and 417 PBP types observed in Datasets 1 and 2. Redline indicates the fitted line of a linear regression. R^2 estimation of the linear regression are shown on top.

Figure S4

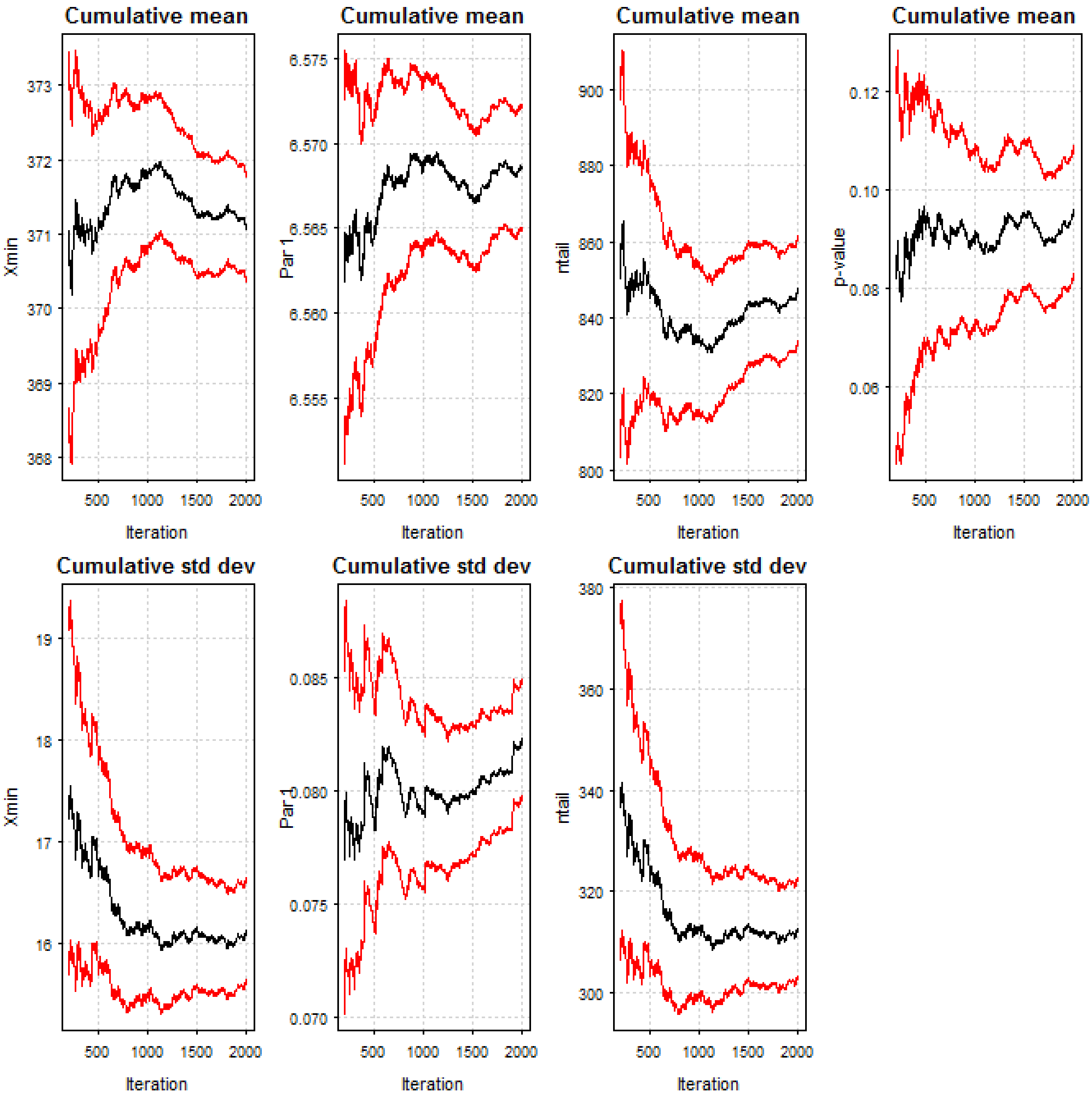

Figure S4. Results from the bootstrap procedure for the power law model. The dashed-lines give approximate 95% confidence intervals.
